# Direct Resin Composite and Lithium Disilicate Endocrowns in Severely Compromised Endodontically Treated Molars: A Two‐Year Randomized Controlled Trial

**DOI:** 10.1111/iej.70232

**Published:** 2026-07-27

**Authors:** Maurits C. F. M. de Kuijper, Carline R. G. van den Breemer, Yvonne C. M. de Waal, Lieke Lanjouw, Mutlu Özcan, Marco S. Cune, Marco M. M. Gresnigt

**Affiliations:** ^1^ Department of Restorative Dentistry and Biomaterials Center for Dentistry Orthodontics, The University of Groningen, University Medical Center Groningen Groningen the Netherlands; ^2^ Department of Special Dental Care Martini Hospital Groningen the Netherlands; ^3^ Department of Epidemiology University Medical Center Groningen, University of Groningen Groningen the Netherlands; ^4^ Center for Dental Medicine, Clinic of Masticatory Disorders and Dental Biomaterials The University of Zurich Zurich Switzerland; ^5^ Department of Oral‐Maxillofacial Surgery, Prosthodontics and Special Dental Care St. Antonius Hospital Nieuwegein Nieuwegein the Netherlands

**Keywords:** composite resins, dental materials, dental porcelain, dental pulp cavity, endocrown, nonvital tooth, patient reported outcome measures

## Abstract

**Aim:**

This randomized controlled superiority trial aimed to compare the clinical performance (restoration survival, clinical quality, and patient satisfaction) of direct resin composite and lithium disilicate endocrown restorations in structurally compromised endodontically treated molars. The null hypothesis stated that there would be no significant difference in clinical performance after 2 years.

**Methodology:**

This was a prospective, randomized superiority controlled trial (ClinicalTrials.gov: NCT06934460). A total of 102 asymptomatic, endodontically treated, and severely structurally compromised molars referred to a university clinic for restorative treatment were included. Teeth were randomly assigned to receive either a direct microhybrid resin composite restoration (*n* = 51) or a lithium disilicate endocrown in conjunction with immediate dentine sealing (*n* = 51). Restoration quality was assessed clinically using Fédération Dentaire International (FDI) criteria at 1 week and at 3, 6, 12, and 24 months. The primary outcome measure was restoration survival (FDI score ≤ 4). Secondary outcomes included abutment tooth survival (extraction of the tooth, regardless of the cause) and abutment tooth success (defined as the absence of tooth extraction, endodontic pathology, or the need for non‐surgical or surgical endodontic retreatment). Patient satisfaction was assessed using a questionnaire over the first year. Kaplan–Meier estimates were used for survival analysis. Restoration quality and satisfaction were compared using Mann–Whitney *U*, Chi‐squared, and Friedman's ANOVA with Bonferroni corrections (*α* = 0.05).

**Results:**

Cumulative restoration survival rates were 98.0% (95% CI: 87.0%–100.0%) and 100.0% for direct resin composite and lithium disilicate endocrowns respectively (*p* = 0.32). Tooth survival was 100.0% in both groups. Indirect endocrowns showed superior surface lustre, wear resistance, and mucosal health after 24 months. Patient satisfaction was similar in both groups.

**Conclusions:**

Both restoration types performed well in restoring severely structurally compromised endodontically treated molar teeth after 24 months. Lithium disilicate endocrowns showed better performance in surface lustre, wear resistance, and mucosal health. However, these qualitative differences did not translate into differences in patient satisfaction, indicating that both restorative options were perceived as equally acceptable by patients in the short‐term.

## 1 Introduction

The prognosis of the endodontically treated tooth depends on multiple factors that can be categorized into operator‐, patient‐, tooth‐ or technical‐related factors (Ng et al. 2010, 2011; Laske et al. 2019; de Kuijper, Meisberger, et al. 2021).

Among the most important tooth‐related factors influencing the survival of the restoration following endodontic treatment is the amount of remaining coronal dentine (Nagasiri and Chitmongkolsuk 2005; Naumann et al. 2005; Signore et al. 2009; Ferrari et al. 2012). Preservation of at least one coronal wall significantly reduces the failure risk as compared to no walls (Hazard Ratio = 0.3 [95%: 0.2–0.7; *p* = 0.00]) (Ferrari et al. 2012). In accordance to this, endodontically treated molars restored with direct resin composites have a reported survival rate of less than 50% after 5 years, except for teeth with a substantial amount of coronal dentine (78%) (Nagasiri and Chitmongkolsuk 2005).

With respect to the technical‐related factors, the coronal restoration is considered a key determinant for the survival and success of the endodontically treated tooth (Ng et al. 2010; Gillen et al. 2011). Within this context, a wide range of coronal restorative options is available, such as a direct resin composite restoration or an indirect restoration made of indirect resin composite, glass–ceramic, high performance ceramic or metal‐ceramic material. In the past, indirect restorations on endodontically treated teeth were often supported by a post and core build‐up. However, this requires a post preparation, which further weakens the tooth (Peroz et al. 2005). Current perspective is that only in cases of extensively compromised endodontically treated teeth with very little remaining coronal tooth structure, the use of a post may be indicated to provide retention to the restorative material (Peroz et al. 2005; Ferrari et al. 2012). Endocrowns appear a viable alternative to the traditional post and core build‐up in structurally compromised molar teeth (Bindl and Mörmann 1999; Otto and Mörmann 2015; Al‐Dabbagh 2021). Endocrowns are monolithic restorations that extend into the pulp chamber of endodontically treated teeth with severe loss of coronal hard tissue. This type of restoration functions particularly well in endodontically treated molar teeth, since their wide pulp chamber provides a large area for adhesion of the restoration (Bindl et al. 2005; Belleflamme et al. 2017). When fabricated from a glass–ceramic or indirect composite, adhesive cementation results in the preservation of tooth structure, because retention does not rely on macro‐geometry or post and core build‐ups. This may contribute to the durable restoration of endodontically treated molar teeth.

In the case of structurally compromised molar teeth with little remaining coronal structure that require restoration, the clinician and patient have to consider treatment alternatives in relation to costs and durability. Indirect restorations are generally associated with higher treatment costs as compared to direct resin composite restorations. The possible alternative could be a directly fabricated resin composite restoration. This can be a clinical challenge for the operator, since it often proves difficult to provide proper marginal adaptation and to adequately restore anatomical form directly in the oral environment. Up to now, there is a lack in randomized controlled trials that compare the two types of restorative modalities and limited evidence suggests no difference in clinical performance between direct resin composites and conventionally cemented porcelain‐fused‐to‐metal crowns after 3 years (de Kuijper et al. 2023). Besides the clinical performance, patient preference also plays a major role in the selection of treatment, as part of patient‐centred care (Mead and Bower 2000; Reissmann 2019; Leles et al. 2022). Patient satisfaction has increasingly become an important outcome measure, as it reflects the patient's perception of function, comfort, and overall treatment acceptability, and may influence restorative decision‐making alongside clinical performance (Reissmann 2019; Leles et al. 2022).

Evidence directly comparing the clinical performance of these two restoration types remains limited. Moreover, data incorporating patient‐reported outcomes in this specific clinical scenario are scarce (Mannocci et al. 2002; Skupien et al. 2016; Bijelic‐Donova et al. 2022; Elhaddad et al. 2024). In addition to traditional clinical outcomes, patient‐reported outcome measures have gained increasing importance in endodontology and restorative dentistry, as they reflect the patient's perspective on treatment success and overall satisfaction. Furthermore, these outcome measures are recognized as a core outcome domain in the S3‐level clinical practice guidelines for the restoration of endodontically treated teeth (Duncan et al. 2023). Therefore, the objective of this randomized controlled trial was to compare the clinical performance of direct resin composite and lithium disilicate endocrown restorations after 24 months of service and patient satisfaction after 12 months. The null hypothesis stated that there would be no difference in the clinical performance or patient satisfaction between the two treatment modalities.

## 2 Material and Methods

### 2.1 Subject Population and Tooth Selection

This randomized, controlled superiority clinical trial received approval from the Medical Ethical Committee of University Medical Center Groningen (NL53678.042.15) and was registered at clinicalTrials.gov (NCT06934460). Patient recruitment took place at the Center for Dentistry Orthodontics, University Medical Center Groningen, between December 2015 and February 2023. Written informed consent was obtained from all participants.

All patients were referred to single university‐based restorative dentistry clinic for restoration of an endodontically treated molar. Endodontic treatment of the included molars had been performed either at the same institution or previously by external general dental practitioners or endodontic specialists prior to referral. Eligible participants were adults (≥ 18 years) with a heavily restored endodontically treated molar (upper or lower) requiring a new restoration. Inclusion criteria required the tooth to be asymptomatic on percussion or palpation, without any suspicious radiographic radiolucency (Peri‐Apical Index (PAI)‐score of 1–3 (Orstavik et al. 1986)). In the absence of a recent radiograph or when the coronal seal was inadequate, a periapical radiograph was obtained to evaluate the quality of the endodontic treatment. Periapical radiographs were assessed for the length and homogeneity of the root canal filling, the presence of visible voids or untreated canals, the quality of the coronal seal, and the periapical status using the Periapical Index (PAI). Further eligibility requirements included the presence of at least a three‐surface defect with 1 or no remaining walls, the necessity for cusp coverage, a minimum pulp chamber height of 2 mm, probing pocket depths of ≤ 5 mm, and the feasibility of rubber dam isolation. A remaining wall was defined as any buccal, palatal/lingual, mesial, or distal supragingival tooth structure anticipated to remain following preparation, with a minimum height of 4 mm. Teeth with clinical symptoms, a PAI‐score of ≥ 4, furcation involvement or lacking an opposing antagonist were excluded. A maximum of two molar teeth per patient could be enrolled in the study. Participants received all restorations at a reduced fee, with no difference in costs between the direct or indirect endocrown.

At the initial consultation, patients were evaluated for caries risk based on their history of restorations placed due to caries in the past 2 years. Those with no prior restorations were classified as low risk, individuals with a single restoration were considered medium risk, and patients who had received two or more restorations due to caries within the past 2 years were categorized as high risk. Additionally, parafunctional habits were recorded when patients reported tooth grinding or clenching, or if wear patterns were observed during the intraoral examination. After inclusion, patients were randomized to receive either a direct micro‐hybrid resin composite or a lithium disilicate endocrown.

Blinding was not feasible due to the distinct technical characteristics of the two treatment approaches. All operative procedures were carried out by a single practitioner (Maurits C.F.M. de Kuijper) at the Center for Dentistry Orthodontics, University Medical Center Groningen. This randomized controlled trial has been written according to Preferred Reporting Items for RAndomized Trials in Endodontics (PRIRATE) 2020 guidelines (Nagendrababu et al. 2020). The PRIRATE flow diagram (Figure 1) provides an overview of the inclusion and allocation process.

**FIGURE 1:**
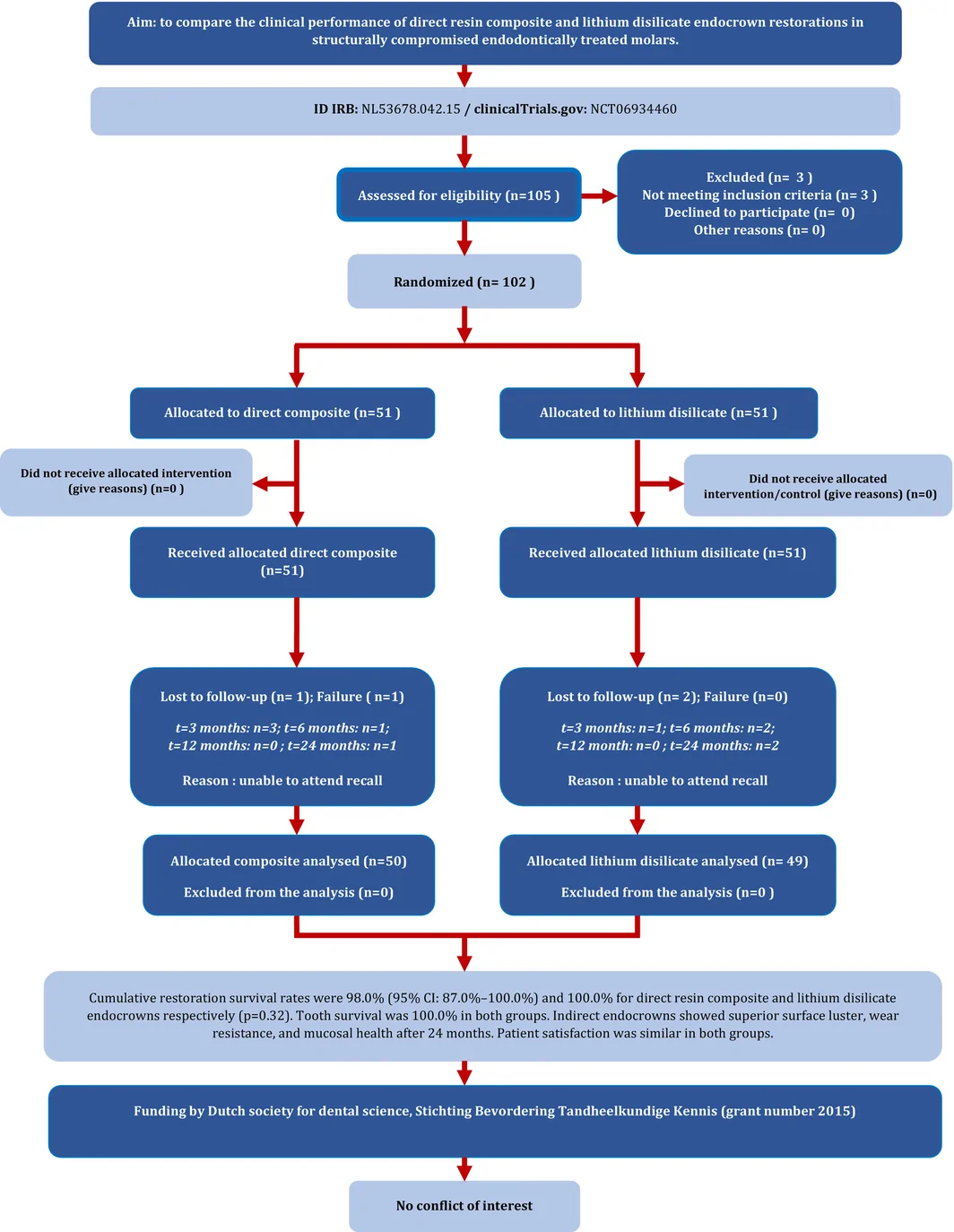
PRIRATE flow chart. From: Nagendrababu et al. (2020) https://doi.org.10.1111/iej.13294. For further details, visit: http://pride‐endodonticguidelines.org/prirate.

### 2.2 Restorative Procedures

A summary of the materials used in this study is provided in Table 1. All restorative procedures were carried out using 3.5× magnification loupes (ExamVision; Rotterdam, the Netherlands). Prior to restoration, margin preparation was performed while preserving the central resin composite to prevent contamination of the endodontic treatment. When required, a gingivectomy was conducted to facilitate proper rubber dam placement. Following isolation, the pulp chamber was prepared, and any exposed gutta‐percha was disinfected using 3% sodium hypochlorite before being sealed with a resin‐modified glass ionomer (Vitre‐bond; 3M ESPE; St. Paul, USA).

**TABLE 1:** Overview of the materials used in this study.

Product name	Type	Manufacturer	Composition
IPS e.max CAD	Lithiumdisilicate	Ivoclar	Silica, lithium oxide, magnesium oxide, lithium oxide, potassium oxide, aluminium oxide, phosphorus pentoxide, other oxides
Clearfil AP‐X Posterior	Micro‐hybrid composite	Kuraray	BisGMA, TEGDMA, barium glass particles, silica, colloidal silica, silicon dioxide
Ultra‐Etch	Phosphoric acid etch	Ultradent	35% phosphoric acid
Optibond FL	Adhesive resin	Kerr	Primer: HEMA, GPDM, PAMA, ethanol, water, CQ, BHT. Adhesive: Bis‐GMA, HEMA, GDMA, CQ, ODMAB, filler (fumed silica, barium aluminoborosilicate glass, disodium hexafluorosilicate), coupling factor A174
Enamel Plus HFO UD2	Micro‐hybrid composite	Micerium	Monomer matrix: Diurethandimethacrylate, Iso‐propyliden‐bis 2(3)‐hydroxy‐3(2)‐4(phenoxy)(propyl)‐bis(methacrylate)(Bis‐GMA), 1,4‐Butandioldimethacrylate. Fillers: highly dispersed silicone dioxide
Vitrebond	Resin modified glass ionomer	3M	
ESPE‐Sil	Silane coupling agent	3M ESPE	3‐methacryloxypropyl‐trimethoxy silane, ethanol, ethyl alcohol
Monobond Plus	Silane coupling agent	Ivoclar	1% 3‐methacryloxypropyl‐trimethoxy silane, ethanol
CoJet‐Sand	Silica‐coated air‐abrasion powder	3 M ESPE	Aluminium trioxide particles coated with silica, particle size: 30 μm
IPS Ceramic Etching Gel	Hydrofluoric acid	Ivoclar	5% hydrofluoric acid
Liquid strip—transparent	Glycerine gel	Ivoclar	Glycerol

#### 2.2.1 Direct Resin Composite Restorations

Enamel and dentine surfaces were etched using 35% phosphoric acid (Ultra‐etch; Ultradent; Cologne, Germany) for 30 and 15 s respectively. The adhesive system (Optibond FL; Kerr; Orange, USA) was applied following the manufacturer's instructions and polymerized for 20 s using a high‐power curing device (Bluephase 20i—1200 mW/cm^2^; Ivoclar; Schaan, Liechtenstein). Prior to curing, light intensity was verified with a radiometer (Bluephase Meter II; Ivoclar).

To assess the amount of remaining tooth structure, a digital impression was taken using an intraoral scanning system (Cerec Omnicam; Dentsply Sirona; Bensheim, Germany). A highly filled micro‐hybrid resin composite (Clearfil AP‐X Posterior; Kuraray Noritake; Okayama, Japan) was applied incrementally in 2‐mm layers, each cured for 20 s. The buccal and lingual walls were initially shaped either freehand or with the aid of a circular matrix band (AutoMatrix; Dentsply). Sectional matrices and separation rings (Palodent V3 Ring; Dentsply Sirona) were then positioned to restore the proximal contours. Final finishing and polishing were performed using rubber polishers (Brownies/Greenies; Shofu). A typical clinical example of a tooth restored with a direct microhybrid resin composite endocrown is presented in Figure 2.

**FIGURE 2:**
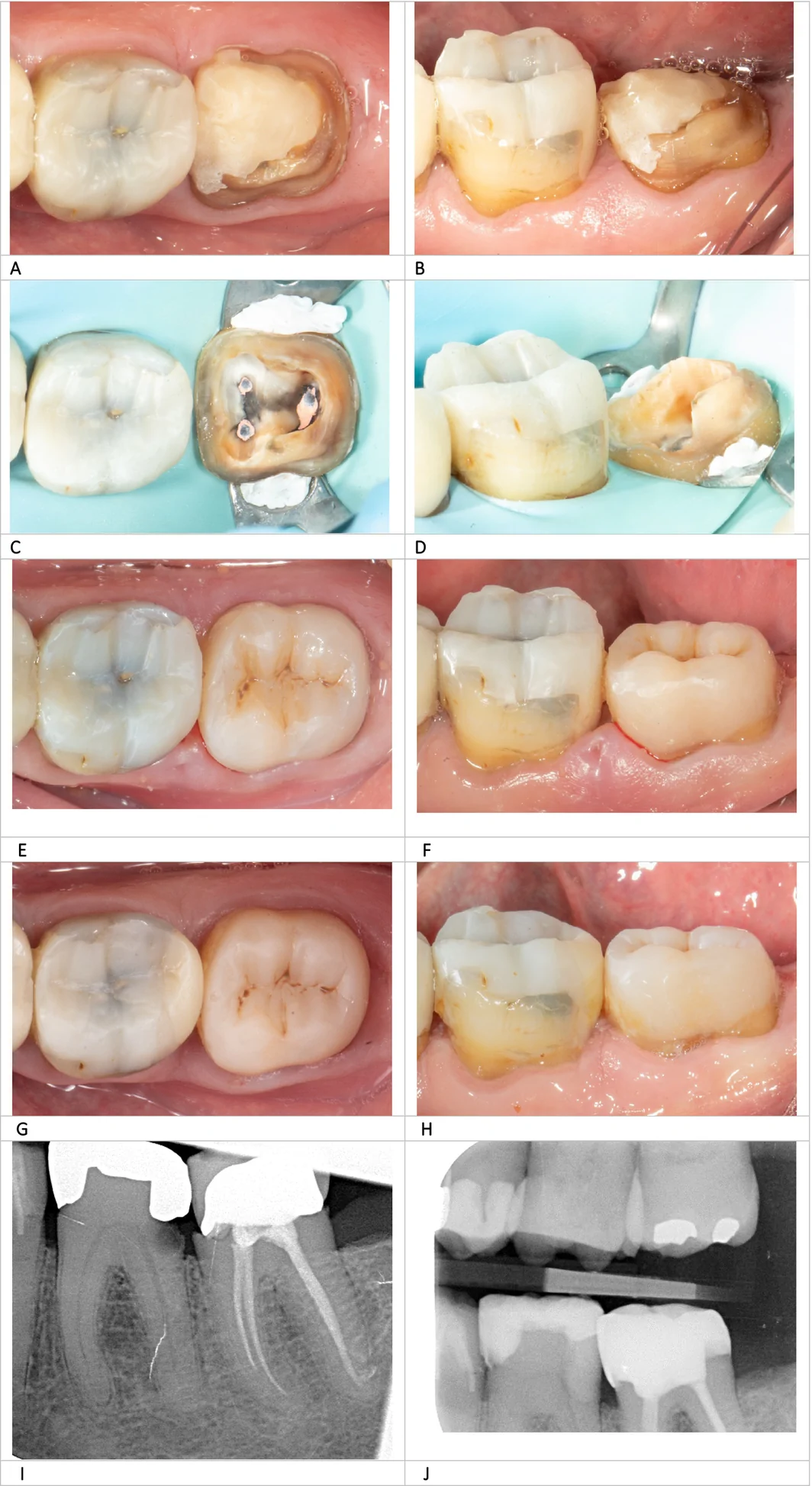
Typical clinical example of a molar tooth (37) restored with a direct microhybrid resin composite endocrown. Occlusal (A) and buccal view (B) of the initial situation. Occlusal (C) and buccal (D) view after removal of the resin composite, decay, and fracture lines. Occlusal (E) and buccal (F) view 1 week after the resin composite restoration. Occlusal (G) and buccal (H) view after 24 months. Intraoral radiographs before (I) and after (J) treatment.

#### 2.2.2 Lithium Disilicate Endocrowns

After preparation, dentine surfaces were sealed using Immediate Dentine Sealing (IDS). The dentine was etched with phosphoric acid for 15 s, rinsed thoroughly with a water spray, and conditioned with a primer (Optibond FL Primer; Kerr) for 20 s using a rubbing motion. After suction‐drying, a bonding agent (Optibond FL Adhesive; Kerr) was applied and light‐cured for 20 s. To eliminate undercuts, a flowable resin composite (Tetric EvoFlow; Ivoclar) was placed. In cases of pronounced undercuts, an additional highly filled microhybrid resin composite resin was used. The final composite layer was coated with glycerin gel (Liquid Strip; Ivoclar) and subsequently light‐cured for 40 s. Enamel margins were refined using a red‐ring diamond bur at low speed. The prepared tooth was then scanned with an intraoral scanner, and a provisional restoration (ProTemp 4; 3M ESPE) was fabricated and cemented using a temporary cement (Durelon; 3M ESPE).

The digital scan data was sent to a dental milling center for the fabrication of a lithium disilicate endocrown (IPS e.max CAD HT; Ivoclar). A single dental technician performed the finishing process, applying staining and glazing according to the manufacturer's guidelines.

Two weeks post‐preparation, the provisional restoration was removed, rubber dam isolation was established, and the fit of the definitive restoration was evaluated. The intaglio surface of the endocrown was conditioned with 4.9% hydrofluoric acid (IPS Ceramic Etch; Ivoclar) for 20 s, followed by ultrasonic cleaning in distilled water for 5 min. A silane coupling agent (Monobond Plus; Ivoclar) was applied for 60 s, followed by the application of a bonding agent (Optibond FL Adhesive). The IDS layer was silica‐coated (CoJet; 3M ESPE) for 3 s until a matte surface was achieved. Enamel margins were etched with 35% phosphoric acid, and the IDS surface was conditioned with silane for 60 s before a bonding agent (Optibond FL Adhesive) was applied to the entire preparation. For delivery, a heated light‐curing micro‐hybrid resin composite (Enamel Plus HFO UD2; Micerium; Avegno, Italy) was applied to the preparation, and the endocrown was seated under constant pressure while excess material was carefully removed from the margins. Polymerization was performed for 30 s per side (occlusal, buccal, and lingual), followed by the application of glycerin gel to the margins and an additional curing cycle of 60 s per side (de Kuijper, Ong, et al. 2021). A typical clinical example of a tooth restored with a lithium disilicate endocrown is presented in Figure 3.

**FIGURE 3:**
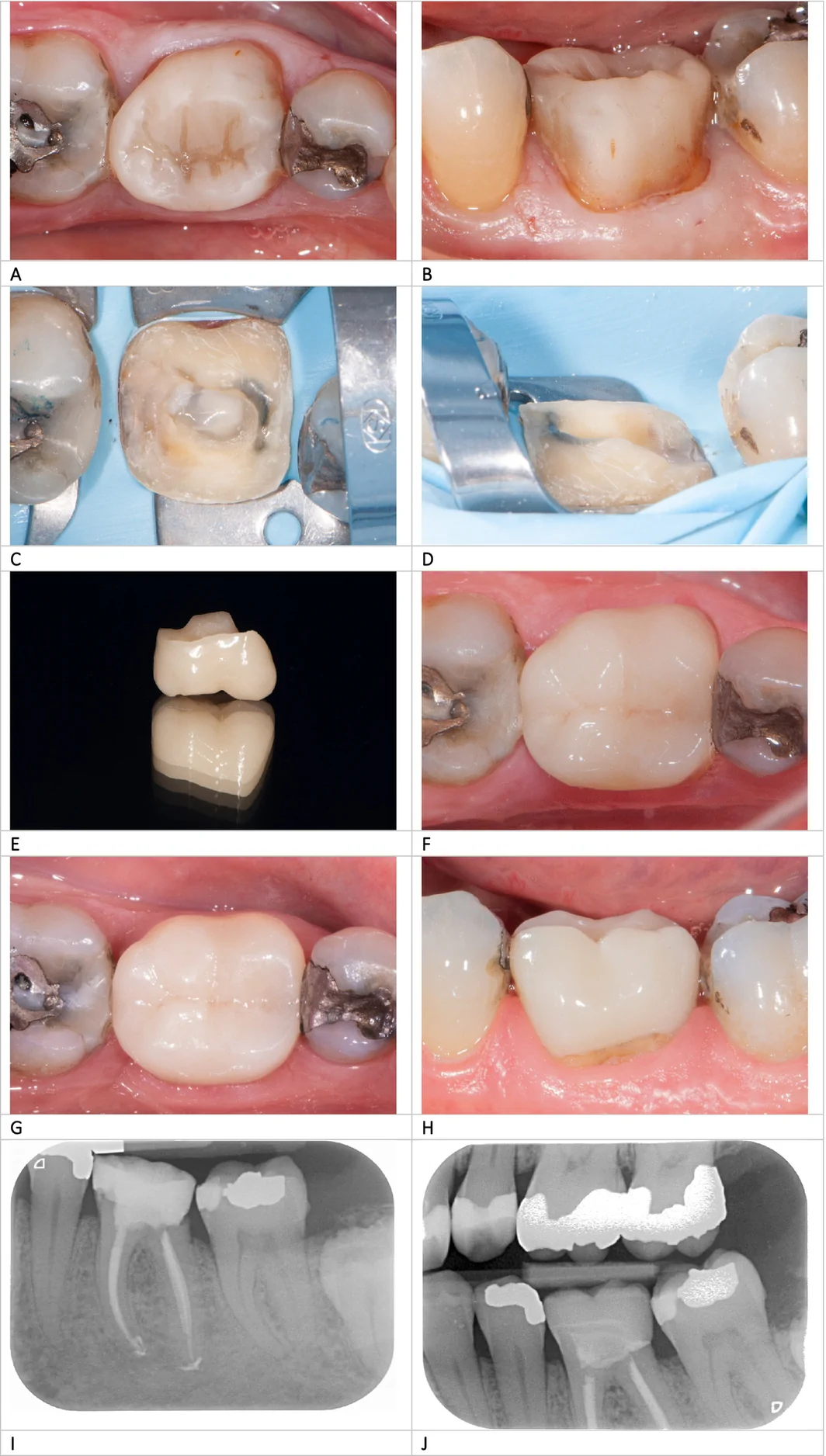
Typical clinical example of a molar tooth (36) restored with a lithium disilicate endocrown. Occlusal (A) and buccal view (B) of the initial situation. Occlusal (C) and buccal (D) view after removal of the resin composite and application of the Immediate Dentine Sealing (IDS). Lithium disilicate endocrown (E) and occlusal view (F) after 1 week. Occlusal (G) and buccal (H) view after 24 months. Radiographs before (I) and after (J) treatment.

### 2.3 Follow‐Up and Study Parameters

Baseline characteristics were noted at patient level (caries risk and parafunctional habits), operator level (endodontic treatment performed in a university setting, general or endodontic specialist practice) or at tooth level (presence of radiolucency before endodontic treatment, type of endodontic treatment, number of remaining walls post‐preparation, the dynamic occlusion after restoration: group function or no lateral contacts, terminal tooth in the arch or the number of adjacent teeth, and surface area of the preparation). Restorations were assessed clinically at 1 week, and 3, 6, 12, and 24 months post‐placement. Their clinical performance was evaluated using the Fédération Dentaire International (FDI) clinical criteria, which provide a standardized framework to assess the clinical performance of dental restorations (Hickel et al. 2010). The FDI criteria consist of aesthetic, functional, and biological domains, with each parameter scored on a five‐point ordinal scale ranging from clinically excellent (score 1) to clinically poor and replacement necessary (score 5). In the present study, surface lustre, staining, colour match, and anatomical form were evaluated under the aesthetic domain. Fracture, marginal adaptation, wear, approximal anatomical form, radiographic examination, and patient view were assessed as part of the functional properties, whereas the presence of caries, tooth integrity, the periodontal response, the condition of the surrounding mucosa, and oral and general health status were evaluated under the biological domain. To further document the condition of the endodontically treated tooth, standardized light photographs were captured from occlusal, lingual, and buccal perspectives using a Digital Single‐Lens Reflex (DSLR) camera (Canon 80D, 100 mm F2.8 macro; Canon Inc., Tokyo, Japan). All assessments were conducted with 3.5× magnification loupes.

Two experienced independent clinicians (Carline R.G. van den Breemer and Yvonne C.M. de Waal), who were not involved in patient selection or treatment, conducted the clinical evaluations. Prior to assessment, they underwent calibration through a web‐based training program in 2015 (www.e‐calib.info). Due to the distinct nature of the treatment modalities, blinding was not feasible. Percentage agreement was calculated for 24 randomly selected cases (12 composite resin and 12 lithium disilicate endocrowns). These cases were additionally evaluated by both independent clinicians based on the photographs after 24 months on surface lustre, staining, colour match, anatomy, fracture, adaptation, wear, baseline radiograph, caries, integrity and mucosa. The FDI‐score was aggregated in 3 scores: scores 1–3 (clinically acceptable), 4 (shortcomings) and 5 (unacceptable) and absolute interoperator agreement was calculated.

The primary outcome measure was restoration survival, defined as restorations receiving an FDI score between 1 and 4 (ranging from clinically excellent to clinically acceptable). A restoration was considered a failure if the tooth was extracted due to reasons that could be attributable to the restoration, such as caries or a vertical root fracture, or if it received an FDI score of 5 (clinically unsatisfactory). An exception was made in cases where periapical pathology was radiographically evident but the restoration remained functionally intact. If no endodontic or surgical retreatment was required, such restorations were still considered to have survived. Secondary outcomes included abutment tooth survival (with failure defined as extraction of the tooth, regardless of the cause), abutment tooth success (failure defined as tooth extraction, the presence of endodontic pathology, or the necessity for (non‐)surgical endodontic retreatment), restoration quality (according to the FDI criteria) and patient satisfaction. To assess patient satisfaction, a questionnaire incorporating Visual Analogue Scales (VAS) was administered during recall visits in the first 12 months (see Table 2) (van den Breemer, Gresnigt, et al. 2019). Patient satisfaction was assessed during the first year to capture early treatment acceptance while several patient‐related aspects such as comfort and functional complaints were additionally captured within the functional and biological domains of the FDI evaluation criteria at subsequent follow‐up visits, thereby limiting additional questionnaires and reducing patient burden at later follow‐up visits. To quantify the surface area of a prepared molar, Blender (version 2.82.20.0) was utilized as the 3D modelling and analysis software. Vertices of the prepared surface were selected, duplicated and separated into a new object. The 3D model was imported as an STL file and scaled correctly within the Metric unit system (millimetres). The 3D Print Toolbox Add‐on was activated, and the total surface area (mm^2^) was determined using the ‘Area & Volume’ function.

**TABLE 2:** Patient questionnaire and VAS‐scales.

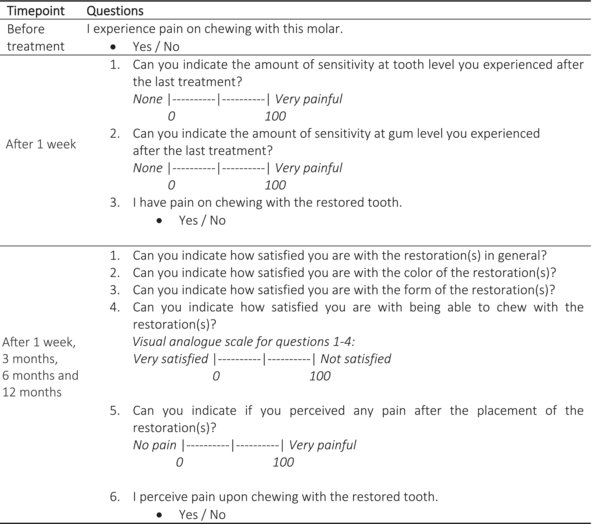

### 2.4 Sample Size and Randomization

The required sample size was determined using GPower (version 3.1.9.2), based on an estimated survival rate of 87.1% for glass–ceramic endocrowns (Bindl et al. 2005). To detect a clinically relevant survival difference of 25% between groups, a sample size calculation was conducted with a two‐tailed alpha level of 0.05, and 80% power, assuming equal group sizes. This estimate of 25% was based on an educated guess. The analysis indicated that a minimum of 51 endodontically treated molars per group needed to be included. Treatment assignment was conducted through minimization, utilizing the online tool ‘Qminim’ (http://rct.mui.ac.ir/q/index.php). The base probability was set at 0.7, and the marginal balance method was applied to compute the imbalance score. Allocation was performed by a researcher (Marco S. Cune) independent of the treating clinician and was conducted at the tooth level, stratified based on three key factors: (1) the number of remaining walls anticipated post‐preparation (one or none), (2) the type of endodontic treatment (primary treatment or retreatment), and (3) the presence or absence of peri‐apical radiolucency on pre‐treatment intraoral radiographs.

### 2.5 Statistical Analysis

Statistical analyses were conducted using IBM SPSS Statistics 24 (SPSS; Chicago, USA). The statistician (Lieke Lanjouw) was blinded to the treatment allocation. Baseline characteristics between the resin composite and lithium disilicate endocrown groups were presented using descriptive statistics at both the patient level (caries risk and parafunctional habits) and the tooth level (presence of radiolucency before endodontic treatment, type of endodontic treatment, number of remaining walls post‐preparation, the dynamic occlusion after restoration, terminal tooth in the arch or the number of adjacent teeth). When applicable, Kaplan–Meier survival estimates were calculated at both restoration and tooth survival and tooth success after 24 months. Differences in survival between treatment groups were assessed using the log‐rank (Mantel–Cox) test. FDI criteria were analysed at baseline, 3, 6, 12 and 24 months using the Mann–Whitney *U*‐test. To compare FDI scores at baseline, 12 and 24 months within each group, Friedman's ANOVA tests were performed. Patient satisfaction across all time points was analysed using chi‐squared, Fisher–Freeman–Halton exact, or Mann–Whitney *U*‐tests, depending on the distribution of the data. Within‐group changes in patient satisfaction over time were evaluated using Friedman's ANOVA, followed by post hoc analysis with Dunn's tests. Bonferroni corrections were applied where necessary to adjust for multiple comparisons. A significance level of 0.05 was set for all statistical tests.

## 3 Results

Between December 2015 and February 2023, a total of 105 endodontically treated molars were screened for eligibility. Two molars were excluded due to vertical root fractures, and one molar required endodontic retreatment due to insufficient root canal obturation and was therefore not included, as the patient did not continue treatment. Consequently, 102 asymptomatic, endodontically treated molars (upper jaw: *n* = 46; lower jaw: *n* = 56) in 85 patients (39 women, 46 men; mean age: 50.64 ± 11.63 years) were included and restored using either direct resin composite (*n* = 51) or lithium disilicate endocrowns (*n* = 51). A total of 17 patients received 2 restorations (15 patients received both types of restorations, 1 patient received 2 resin composite restorations and 1 patient received 2 lithium disilicate endocrowns; Table S1). Of 84 molar teeth, the date of the endodontic (re)treatment was known. Median time between endodontic (re)treatment and the restorative intervention was 56.5 months (minimum‐maximum: 1–242 months; interquartile range: 17–91 months). Of 18 teeth, no exact date could be determined due to insufficient information in the patient records, either because the dentist had retired (*n* = 4) or because the former dentist could not be contacted (*n* = 14). Two teeth received endodontic retreatment before resin composite restoration because of tenderness to percussion in combination with inadequate root canal obturation (*n* = 2). Baseline characteristics are summarized in Table 3.

**TABLE 3:** Distributions of baseline characteristics at patient and tooth level per treatment group. Percentages are presented at the restoration level.

Baseline characteristics	Composite (*n* = 51)	Lithium disilicate (*n* = 51)
Patient level		
Caries risk		
Low	17 (33%)	18 (35%)
Medium	17 (33%)	19 (37%)
High	17 (34%)	14 (28%)
Parafunction		
No	36 (71%)	40 (78%)
Yes	15 (29%)	11 (22%)
Operator level		
Endodontic treatment performed in		
University setting	7 (14%)	9 (18%)
General practice	40 (78%)	35 (69%)
Endodontic specialist practice	4 (8%)	7 (13%)
Tooth level		
Apical radiolucency before endodontic treatment		
Absent	32 (63%)	33 (65%)
Present	19 (37%)	18 (35%)
Type of endodontic treatment		
First	43 (84%)	42 (82%)
Retreatment	8 (16%)	9 (18%)
Periapical Index Score (PAI) before restorative treatment		
1	39 (76%)	34 (66%)
2	11 (22%)	11 (22%)
3	1 (2%)	6 (12%)
Number of walls after preparation		
None	16 (31%)	19 (37%)
1 wall	35 (69%)	32 (63%)
Dynamic occlusion		
No lateral contact during lateral excursions	35 (69%)	25 (49%)
Group function	16 (31%)	26 (51%)
Terminal tooth in the arch		
Terminal tooth	36 (71%)	36 (71%)
No terminal tooth	15 (29%)	15 (29%)
Adjacent teeth		
None	1 (2%)	2 (4%)
1 tooth	16 (31%)	15 (29%)
2 teeth	34 (67%)	34 (67%)
Median surface area (mm^2^)	129.95	115.96
Interquartile range surface area (mm^2^)	37.62	19.75
Minimum‐maximum surface area (mm^2^)	84.86‐178.53	86.28–154.35

### 3.1 Restoration Survival, Abutment Tooth Survival and Success

The cumulative restoration survival rate was 98.0% (95% CI: 87.0%–100.0%) for the direct resin composite restorations and 100.0% for the lithium disilicate endocrowns. No statistically significant difference in restoration survival was observed between the two treatment groups (log‐rank test: *p* = 0.32). Two FDI scores of 5 occurred in the direct resin composite resin group, one due to secondary caries (at 12 months) and one due to a recurrent endodontic sinus tract (at 6 months; see Figures 4 and 5). The secondary caries was treated with a new direct resin composite restoration. For the endodontic sinus tract, in consultation with the patient and the referring dentist, no additional treatment was undertaken, since the tooth already received an endodontic retreatment before enrollment. Retrospectively, the patient reported a prior flare‐up that had not been disclosed during the intake. At both the 12‐ and 24‐month follow‐up evaluations, the restoration remained intact, the patient was asymptomatic, and the sinus tract had fully resolved (Figure 5B). The secondary decay developed after 12 months in a high risk caries patient, in the absence of a proper contact point at baseline and follow‐up. All teeth survived. Abutment tooth success was 98.0% (95% CI: 87.0%–100.0%) for the resin composite restorations and 100.0% for the lithium disilicate endocrowns. The difference between the groups was not statistically significant (log‐rank test, *p* = 0.33).

**FIGURE 4:**
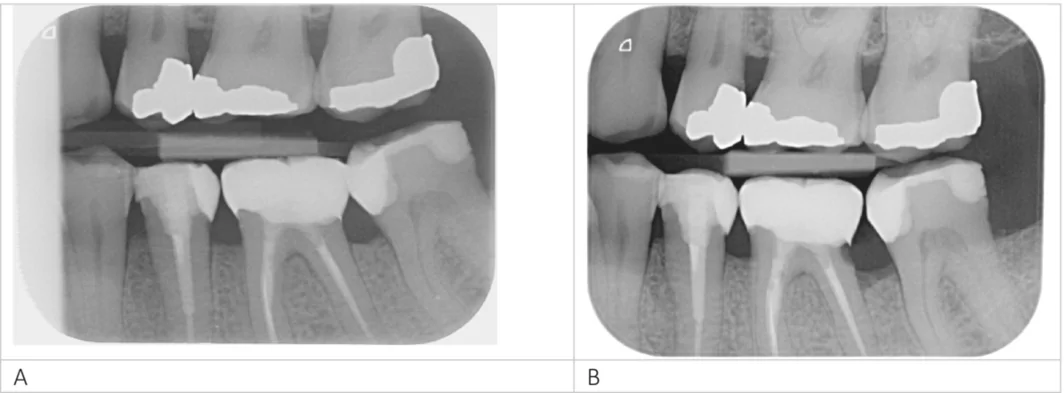
Absolute failure of a resin composite restoration in a high risk caries patient. Radiograph of tooth 36 after restoration (A) and after 12 months (B), showing a radiolucency on the distal aspect, indicating secondary caries. The same patient also presented with a large caries lesion in tooth 46.

**FIGURE 5:**
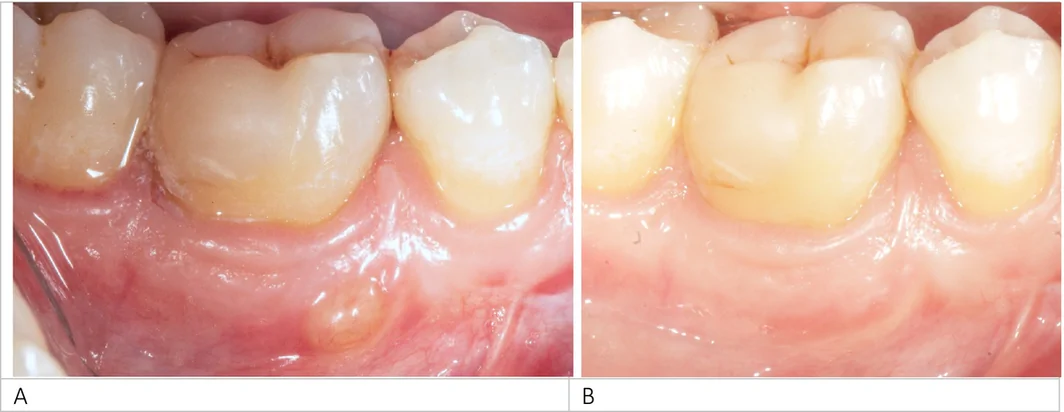
Tooth 36 (resin composite) with an endodontic sinus tract after 6 months (A) that was completely resolved after 24 months without any intervention (B), with the restoration remaining intact.

### 3.2 Restoration Quality

At all assessed time points—baseline (*U* = 2221.50; *p* = 0.00; *r* = 0.72), 3 months (*U* = 2112.00; *p* = 0.00; *r* = 0.75), 6 months (*U* = 2263.00; *p* = 0.00; *r* = 0.82), 12 months (*U* = 2433.50; *p* = 0.00; *r* = 0.84), and 24 months (*U* = 2314.50; *p* = 0.00; *r* = 0.87)—resin composite restorations demonstrated a significantly lower mean rank in surface lustre compared to lithium disilicate endocrowns (Table 4). Although resin composite exhibited superior performance in terms of marginal staining at baseline (*U* = 1147.50; *p* = 0.03; *r* = −0.22), lithium disilicate restorations outperformed resin composite at 6 months (*U* = 1472.50; *p* = 0.01; *r* = 0.25), 12 months (*U* = 1587.50; *p* = 0.02; *r* = 0.22), and 24 months (*U* = 1464.50; *p* = 0.02; *r* = 0.23). In terms of colour match, resin composite achieved significantly better scores at baseline (*U* = 897.00; *p* = 0.00; *r* = −0.30), 6 months (*U* = 889.50; *p* = 0.01; *r* = −0.26), and 12 months (*U* = 1009.50; *p* = 0.03; *r* = −0.22); however, no statistically significant difference was observed at 24 months (*U* = 1193.00; *p* = 0.95; *r* = −0.01). Wear resistance was significantly greater in lithium disilicate endocrowns, with lower wear scores recorded at both 12 months (*U* = 1402.50; *p* = 0.04; *r* = 0.20) and 24 months (*U* = 1396.50; *p* = 0.00; *r* = 0.30). Additionally, mucosal health was significantly better in the lithium disilicate group at 3 months (*U* = 1480.50; *p* = 0.02; *r* = 0.24) and 24 months (*U* = 1530.00; *p* = 0.01; *r* = 0.28). For the resin composite restorations, both surface lustre and colour match showed a statistically significant deterioration at 24 months compared to baseline (*Z* = −3.18, *p* = 0.00, *r* = −0.46 and *Z* = −3.08, *p* = 0.01, *r* = −0.44, respectively). Similarly, surface staining significantly increased at both 12 months (*Z* = −3.54, *p* = 0.00, *r* = −0.51) and 24 months (*Z* = −3.28, *p* = 0.00, *r* = −0.47). There were no significant differences over time for the lithium disilicate endocrowns. After 24 months, contact point strength increased in 24.49% of the resin composite and 22.44% of the lithium disilicate restorations, remained unchanged in 34.69% and 42.86%, and decreased in 40.82% and 34.69%, respectively, with no significant difference between the groups (*p* = 0.11). For one resin composite restoration, one adjacent crown dislodged during follow‐up, resulting in a weaker contact point. Interoperator agreement ranged from 95.8% (caries) to 100% (all other criteria).

**TABLE 4:** Summary of the FDI criteria evaluation at 1 week, at 3, 6 and 12 months.

FDI criteria	Score	Composite					Lithium disilicate				
		1 week	3 months	6 months	12 months	24 months	1 week	3 months	6 months	12 months	24 months
A. Aesthetic properties											
1. Surface lustre	1	14 (27%)	9 (19%)	5 (10%)	5 (10%)	1 (2%)	50 (98%)	47 (94%)	46 (94%)	49 (96%)	45 (92%)
	2	31 (61%)	33 (69%)	38 (76%)	35 (69%)	30 (61%)	1 (2%)	3 (6%)	3 (6%)	2 (4%)	4 (8%)
	3	6 (12%)	6 (12%)	7 (14%)	11 (21%)	18 (37%)	—	—	—	—	—
	4	—	—	—	—	—	—	—	—	—	—
	5	—	—	—	—	—	—	—	—	—	—
2. Staining surface/margin	1	50 (98%)	41 (85%)	35 (70%)	25 (49%)	27 (55%)	44 (86%)	45 (90%)	44 (90%)	37 (73%)	39 (80%)
	2	1 (2%)	6 (13%)	13 (26%)	25 (49%)	17 (35%)	7 (14%)	5 (10%)	5 (10%)	12 (23%)	5 (10%)
	3	—	1 (2%)	2 (4%)	1 (2%)	5 (10%)	—	—	—	2 (4%)	5 (10%)
	4	—	—	—	—	—	—	—	—	—	—
	5	—	—	—	—	—	—	—	—	—	—
3. Colour match and translucency	1	35 (69%)	30 (63%)	31 (62%)	25 (49%)	14 (29%)	22 (43%)	25 (50%)	18 (37%)	17 (33%)	16 (33%)
	2	16 (31%)	17 (35%)	17 (34%)	25 (49%)	31 (63%)	20 (39%)	18 (36%)	25 (51%)	26 (51%)	26 (53%)
	3	—	1 (2%)	2 (4%)	1 (2%)	4 (8%)	9 (18%)	7 (14%)	6 (12%)	8 (16%)	7 (14%)
	4	—	—	—	—	—	—	—	—	—	—
	5	—	—	—	—	—	—	—	—	—	—
4. Aesthetic anatomical form	1	45 (88%)	45 (94%)	47 (94%)	46 (90%)	39 (80%)	45 (88%)	43 (86%)	46 (94%)	47 (92%)	44 (90%)
	2	5 (10%)	—	3 (6%)	4 (8%)	9 (18%)	6 (12%)	7 (14%)	3 (6%)	3 (6%)	5 (10%)
	3	1 (2%)	3 (6%)	—	1 (2%)	1 (2%)	—	—	—	1 (2%)	—
	4	—	—	—	—	—	—	—	—	—	—
	5	—	—	—	—	—	—	—	—	—	—
B. Functional properties											
5. Fracture of material and retention	1	51 (100%)	48 (100%)	48 (96%)	50 (98%)	48 (98%)	51 (100%)	50 (100%)	49 (100%)	51 (100%)	49 (100%)
	2	—	—	2 (4%)	—	—	—	—	—	—	—
	3	—	—	—	1 (2%)	1 (2%)	—	—	—	—	—
	4	—	—	—	—	—	—	—	—	—	—
	5	—	—	—	—	—	—	—	—	—	—
6. Marginal adaptation	1	34 (67%)	28 (58%)	25 (50%)	21 (41%)	20 (41%)	38 (74%)	38 (76%)	30 (61%)	24 (47%)	30 (61%)
	2	16 (31%)	17 (35%)	21 (42%)	28 (55%)	29 (59%)	11 (22%)	10 (20%)	17 (35%)	26 (51%)	15 (31%)
	3	1 (2%)	3 (6%)	4 (8%)	2 (4%)	—	2 (4%)	2 (4%)	2 (4%)	1 (2%)	4 (8%)
	4	—	—	—	—	—	—	—	—	—	—
	5	—	—	—	—	—	—	—	—	—	—
7. Occlusal contour wear	1	51 (100%)	46 (96%)	47 (94%)	47 (92%)	41 (84%)	51 (100%)	50 (100%)	47 (100%)	51 (100%)	49 (100%)
	2	—	2 (4%)	3 (6%)	4 (8%)	5 (10%)	—	—	—	—	—
	3	—	—	—	—	2 (4%)	—	—	—	—	—
	4	—	—	—	—	—	—	—	—	—	—
	5	—	—	—	—	—	—	—	—	—	—
8. Approximal anatomical form contact point/contour	1	9 (18%)	10 (21%)	11 (22%)	10 (20%)	10 (20%)	20 (39%)	18 (36%)	18 (37%)	11 (29%)	12 (24%)
	2	17 (33%)	13 (27%)	11 (22%)	13 (25%)	15 (31%)	12 (24%)	9 (18%)	12 (24%)	13 (25%)	15 (31%)
	3	21 (41%)	18 (38%)	19 (38%)	17 (33%)	14 (29%)	15 (29%)	19 (38%)	14 (29%)	14 (27%)	13 (27%)
	4	3 (6%)	6 (12%)	8 (16%)	10 (20%)	9 (18%)	2 (4%)	2 (4%)	3 (6%)	7 (14%)	7 (14%)
	5	—	—	—	—	—	—	—	—	—	—
	NA	1 (2%)	1 (2%)	1 (2%)	1 (2%)	1 (2%)	2 (4%)	2 (4%)	2 (4%)	2 (4%)	2 (4%)
9. Radiographic examination	1	47 (92%)	—	—	—	6 (12%)	46 (90%)	—	—	—	13 (27%)
	2	4 (8%)	—	—	—	2 (4%)	5 (10%)	—	—	—	—
	3	—	—	—	—	—	—	—	—	—	—
	4	—	—	—	—	—	—	—	—	—	—
	5	—	—	1 (2%)	—	—	—	—	—	—	—
	NA	—	48 (100%)	49 (98%)	49 (100%)	41 (84%)	—	50 (100%)	49 (100%)	51 (100%)	35 (73%)
10. Patient's view	1	43 (84%)	45 (94%)	47 (94%)	45 (88%)	49 (100%)	46 (90%)	48 (96%)	47 (96%)	51 (100%)	48 (98%)
	2	4 (8%)	—	2 (4%)	4 (8%)	—	2 (4%)	1 (2%)	2 (4%)	—	1 (2%)
	3	4 (8%)	3 (6%)	1 (2%)	2 (4%)	—	3 (6%)	1 (2%)	—	—	—
	4	—	—	—	—	—	—	—	—	—	—
	5	—	—	—	—	—	—	—	—	—	—
C. Biological properties											
11. Recurrence of caries	1	51 (100%)	48 (100%)	50 (100%)	50 (98%)	49 (100%)	51 (100%)	50 (100%)	49 (100%)	51 (100%)	49 (100%)
	2	—	—	—	—	—	—	—	—	—	—
	3	—	—	—	—	—	—	—	—	—	—
	4	—	—	—	—	—	—	—	—	—	—
	5	—	—	—	1 (2%)	—	—	—	—	—	—
12. Tooth integrity (enamel crack, tooth fractures)	1	50 (98%)	47 (98%)	49 (98%)	50 (98%)	43 (88%)	51 (100%)	48 (96%)	49 (100%)	48 (94%)	47 (96%)
	2	1 (2%)	1 (2%)	1 (2%)	1 (2%)	6 (12%)	—	2 (4%)	—	3 (6%)	2 (4%)
	3	—	—	—	—	—	—	—	—	—	—
	4	—	—	—	—	—	—	—	—	—	—
	5	—	—	—	—	—	—	—	—	—	—
13. Periodontal response (compared to a reference tooth)	1	9 (18%)	17 (35%)	14 (28%)	13 (26%)	10 (20%)	17 (33%)	18 (36%)	21 (43%)	15 (29%)	16 (33%)
	2	19 (37%)	11 (23%)	12 (24%)	9 (17%)	5 (10%)	18 (35%)	19 (38%)	11 (22%)	18 (35%)	10 (20%)
	3	23 (45%)	20 (42%)	23 (46%)	29 (57%)	34 (70%)	16 (32%)	13 (26%)	17 (35%)	18 (35%)	22 (45%)
	4	—	—	1 (2%)	—	—	—	—	—	—	1 (2%)
	5	—	—	—	—	—	—	—	—	—	—
14. Adjacent mucosa	1	30 (59%)	26 (54%)	25 (50%)	25 (49%)	14 (29%)	37 (73%)	39 (78%)	32 (65%)	30 (59%)	27 (55%)
	2	20 (39%)	21 (44%)	24 (48%)	26 (51%)	34 (69%)	13 (25%)	10 (20%)	17 (35%)	21 (41%)	22 (45%)
	3	1 (2%)	1 (2%)	1 (2%)	—	1 (2%)	1 (2%)	1 (2%)	—	—	—
	4	—	—	—	—	—	—	—	—	—	—
	5	—	—	—	—	—	—	—	—	—	—
15. Oral and general health	1	51 (100%)	48 (100%)	49 (98%)	51 (100%)	49 (100%)	49 (96%)	49 (98%)	49 (100%)	51 (100%)	49 (100%)
	2	—	—	1 (2%)	—	—	1 (2%)	1 (2%)	—	—	—
	3	—	—	—	—	—	1 (2%)	—	—	—	—
	4	—	—	—	—	—	—	—	—	—	—
	5	—	—	—	—	—	—	—	—	—	—

*Note:* Missing recalls composite: *N* = 3 at 3 months, *n* = 1 at 6 months, *n* = 2 at 12 months. At 24 months there was 1 missing recall and 1 restoration failed at 12 months. Missing recalls lithium disilicate: *N* = 1 at 3 months, *n* = 2 at 6 months and *n* = 2 at 24 months.

Abbreviation: NA, not applicable.

### 3.3 Patient Satisfaction

There was no statistically significant difference between the direct resin composite and lithium disilicate endocrowns concerning patient satisfaction after 1 week and 3, 6 and 12 months (all *p* > 0.05, Mann–Whitney *U*‐tests; see Table 5). For direct resin composite restorations, patient satisfaction remained consistent across all time points, with no significant differences detected. However, for lithium disilicate endocrowns, time had a significant impact on two factors: perceived ability to chew (*χ* ^2^[3] = 12.15, *p* = 0.01) and post‐placement pain (*χ* ^2^[3] = 10.53, *p* = 0.02). Post hoc analysis indicated a significantly better score for chewing ability (*p* = 0.05; *r* = −0.37) and reduced pain levels (*p* = 0.05; *r* = −0.38) at 12 months compared to the 1‐week assessment.

**TABLE 5:** Overview of patient satisfaction before treatment and 1 week, 3, 6 and 12 months after treatment.

	Comp	LiDi	p
**Before treatment**			
I experience pain on chewing with this molar	Yes: 3 (6%)	Yes: 0 (0%)	0.24
	No: 48 (94%)	No: 51 (100%)	
**After placement**			
Can you indicate the amount of sensitivity at tooth level you experienced after the last treatment? (VAS, range: none to very painful)	7 (0–73)	5 (0–70)	0.44
Can you indicate the amount of sensitivity at gum level you experienced after the last treatment?	15 (0–91)	14 (0–87)	0.97
I have pain on chewing with the restored tooth	Yes: 6 (12%)	Yes: 4 (8%)	0.74
	No: 45 (88%)	No: 47 (92%)	

Recalls	1 week			3 months			6 months			12 months		
	Comp	LiDi	*p*	Comp	LiDi	*p*	Comp	LiDi	*p*	Comp	LiDi	*p*
General satisfaction	3.5 (0–45)	3 (0–42)	0.93	4 (0–30)	3 (0–29)	0.39	2.5 (0–24)	3 (0–20)	0.69	3 (0–18)	2 (0–25)	0.37
Satisfaction with the colour of the restoration	3 (0–61)	3 (0–35)	0.55	4 (0–46)	3 (0–35)	0.72	2.5 (0–32)	3 (0–28)	0.57	3 (0–22)	2 (0–23)	0.75
Satisfaction with the shape of the restoration	4 (0–55)	3 (0–35)	0.77	4.5 (0–31)	3 (0–31)	0.83	3 (0–30)	3 (0–20)	0.99	3 (0–33)	2 (0–31)	0.89
Satisfaction with the ability to chew with the restoration	3 (0–64)	3 (0–53)	0.80	4 (0–33)	2 (0–39)	0.19	2 (0–28)	2 (0–31)	0.41	2.5 (0–17)	1 (0–16)	0.59
Pain	5 (0–55)	4 (0–72)	0.98	3.5 (0–86)	2 (0–77)	0.95	2.5 (0–34)	3 (0–65)	0.56	1.5 (0–48)	1 (0–35)	0.34
I have pain on chewing with the restored tooth	Yes: 2 (4%); No: 49 (96%)	Yes: 3 (6%); No: 48 (94%)	1.00	Yes: 2 (4%); No: 46 (96%)	Yes: 2 (4%); No: 48 (96%)	1.00	Yes: 1 (2%); No: 49 (98%)	Yes: 0 (0%); No: 49 (100%)	1.00	Yes: 0 (0%); No: 51 (100%)	Yes: 0 (0%); No: 51 (100%)	—

*Note:* For the Visual Analogue Scales (VAS) median scores are presented along with the minimum and maximum values and *p*‐value of the Mann Whitney *U*‐tests. The VAS scores ranged from score 0 (no pain or very satisfied) to 100 (very painful or not satisfied). For the dichotomized variables, *p*‐value of the chi‐squared test or Fisher–Freeman–Halton Exact test with Bonferroni corrections are presented.

Abbreviations: Comp, resin composite; LiDi, lithium disilicate; *p*, *p*‐value.

## 4 Discussion

The aim of this randomized controlled trial was to evaluate and compare the clinical performance of direct microhybrid resin composite and lithium disilicate endocrown restorations in structurally compromised molar teeth. The findings of the present study demonstrate high survival rates for both direct micro‐hybrid resin composite restorations and lithium disilicate endocrowns after 24 months, with cumulative survival rates of 98.0% and 100.0%, respectively. While direct comparisons with other literature should be interpreted with caution due to differences in case selection, these findings align with earlier clinical trials reporting favourable outcomes for both direct and indirect restorations in endodontically treated posterior teeth on the short term (Mannocci et al. 2002; Cagidiaco et al. 2007; Skupien et al. 2016; Bijelic‐Donova et al. 2022; de Kuijper et al. 2023; Elhaddad et al. 2024; Abu‐Awwad et al. 2025). To the author's knowledge, only 2 randomized controlled trials compared direct resin composite to indirect endocrowns (Bijelic‐Donova et al. 2022; Elhaddad et al. 2024). In a 4‐year randomized controlled trial, short‐fibre reinforced resin composite restorations performed equally to leucite‐reinforced feldspathic endocrowns in terms of restoration survival (Bijelic‐Donova et al. 2022). However, direct resin composite restorations required more maintenance over time, including polishing after minor chipping (*n* = 1), repair due to secondary caries (*n* = 2), and intervention for loss of approximal contact (*n* = 2), whereas only one glass–ceramic restoration required polishing. Similarly, in a 1‐year randomized controlled trial by Elhaddad et al., all direct and CAD/CAM composite endocrowns remained intact and demonstrated comparable restoration quality (Elhaddad et al. 2024). The sample size of both studies was small and Elhaddad et al. experienced a considerable loss to follow‐up (18.8%), which may affect the generalizability of the results. In the present study, the loss to follow‐up in both treatment arms was limited to 3.92%. Other randomized controlled trials compared full contour crowns with direct resin composite restorations on endodontically treated posterior teeth after a follow‐up period of 3 years, with no difference in restoration survival (Mannocci et al. 2002; Abu‐Awwad et al. 2025).

Retrospective studies too have compared both treatment modalities (Aquilino and Caplan 2002; Dammaschke et al. 2004, 2013; Pratt et al. 2016; Suksaphar et al. 2018; Jirathanyanatt et al. 2019). However interpretation is often limited due to clustering of both amalgam and composite restorations, heterogenous case selection, difference in residual tooth structure and a high risk of selection bias (Aquilino and Caplan 2002; Dammaschke et al. 2004, 2013; Pratt et al. 2016). Frequently endodontically treated teeth restored with direct resin composites had a poorer prognosis than indirectly restored teeth, representing an important confounding factor (Suksaphar et al. 2018; Jirathanyanatt et al. 2019; de Kuijper et al. 2023). To address these limitations, the present randomized controlled trial employed a minimization protocol to ensure balanced allocation of molars between treatment groups based on the number of remaining walls expected after preparation, thereby better controlling for the amount of residual tooth structure in both arms. Achieving homogeneity in baseline characteristics is a known challenge in clinical research, particularly as the extent of remaining tooth structure cannot always be accurately predicted preoperatively due to factors such as hidden fractures, caries, or compromised wall thickness. To address this, photographs and intraoral scans were obtained following preparation to assess the number of residual walls. Upon review of the baseline characteristics, both treatment groups were found to be comparable with respect to the number of remaining walls (Table 3). Based on the limited available evidence, short‐term restoration survival appears comparable between direct and indirect restorations on endodontically treated posterior teeth. However, direct resin composite restorations may require more maintenance over time. The resin composite restorations demonstrated significantly lower surface lustre from the outset and consistently throughout the follow‐up period. This outcome is inherent to the material properties of the microhybrid resin composite used, in contrast to the lithium disilicate, which was glazed and polished extraorally to achieve a high‐gloss finish comparable to natural enamel. The comparatively rougher surface texture of the composite resin may also account for the observed increase in surface staining at the end of the observation period relative to baseline. Conversely, the aesthetic advantages associated with indirect restorations may hold limited clinical relevance in the posterior region, where visual appearance is typically less of a concern. Long‐term follow‐ups are needed to compare both treatment modalities over time.

Restorations that received an FDI score of 4 were primarily related to contact point deficiencies. Achieving adequate proximal contacts is a known technical challenge in severely structurally compromised molars, particularly for direct restorations, due to extensive cavity geometry and reliance on operator skill. At baseline, weak contact points (FDI score 3 or 4) were observed in 46% of the resin composite restorations and 33% of the lithium disilicate endocrowns. Notably, weak contact points were observed in a substantial proportion of indirect restorations shortly after placement, despite verification prior to cementation. The increase in deficient contact points over time was seen in both treatment groups and is likely multifactorial. Firstly, significant alterations in the occlusal anatomy were made because all molar teeth were severely compromised, which may have led to changes in occlusal contacts, potentially resulting in tooth migration due to orthodontic forces. Secondly, a change in the position or restoration of the adjacent teeth could be an explanation. Some neighbouring teeth did not have an antagonistic tooth, which might have resulted in overeruption. Furthermore, one adjacent crown dislodged during follow‐up, resulting in a weaker contact point. A third explanation could be a measurement error. However, due to the use of metal strips (Matrix EX; Deppeler SA, Rolle, Switzerland) to measure the quality of the contact point, this is unlikely. In addition, a gingivectomy was often required to achieve proper isolation. Periodontal remodelling following the procedure may also have contributed to changes in tooth position over time. A potential risk for future contact point changes is the need for reintervention on adjacent teeth due to the elevated caries and parafunctional risk commonly observed in this patient population.

The primary causes of failure for endocrowns reported in the literature are debonding and fracture of the glass–ceramic material (Bindl et al. 2005). Within the limited 24‐month observation period of the present study, no instances of debonding or fracture were observed. In vitro studies comparing direct and indirect endocrowns on molar teeth, show that both treatment modalities result in a tooth‐restoration complex that can withstand the masticatory forces (Dartora et al. 2019; de Kuijper et al. 2019). In the current randomized controlled study, lithium disilicate endocrowns were cemented using a microhybrid resin composite, as was described by Bindl et al. (Bindl et al. 2005). In vitro studies have demonstrated that adequate curing is possible through high‐translucency lithium disilicate when using high‐intensity curing lights combined with extended curing times (Gregor et al. 2014; Kameyama et al. 2015; de Kuijper, Ong, et al. 2021). In Bindl et al.'s (2005) study, a debonding rate of 16% was reported. Notably, the authors attributed these failures to the resin‐dentine interface, as the composite resin on the intaglio surface of the endocrowns was found to be fully polymerized. This highlights the persistent challenge of achieving a durable bond to dentine (Perdigão 2010). Especially in endodontically treated teeth where restoration margins are often located partially or entirely in dentine due to prior structural loss. To optimize the bond strength to dentine, Immediate Dentine Sealing was applied (Magne et al. 2005; van den Breemer, Özcan, et al. 2019). To the best of our knowledge, there is currently only one other study available having included an IDS layer during the restoration of endocrowns. Belleflamme et al. (2017) reported a failure percentage of 2% due to debondings over a 10‐year period for endocrowns placed in conjunction with an IDS layer. Partial indirect restorations in conjunction with IDS also present with a favourable survival rate, although a distinct clinical benefit of IDS could not be demonstrated in vital molar teeth with partial restorations after an observation period of 3 years (van den Breemer, Cune, et al. 2019; van den Breemer et al. 2021).

Patient‐reported outcomes have increasingly been incorporated in restorative and prosthetic dentistry, but remain scarce in studies evaluating the restoration of endodontically treated posterior teeth. To the authors' knowledge, no previous randomized controlled trial has directly compared patient satisfaction after direct resin composite restorations and lithium disilicate endocrowns in severely structurally compromised endodontically treated molars. In the present study, patient satisfaction during the first year was high and comparable between direct resin composite restorations and lithium disilicate endocrowns, despite observed differences in certain clinical parameters such as surface lustre and wear resistance. Patient satisfaction represents an increasingly important outcome in restorative dentistry, reflecting the patient's perception of function, comfort, and overall treatment acceptability, and supporting shared decision‐making in clinical practice (Reissmann 2019; Leles et al. 2022). Evidence from adjacent restorative fields suggests that material choice might not always influence the impact on the oral‐health‐related quality of life. In patients treated for severe tooth wear, restorative treatment has been associated with improved oral‐health‐related quality of life, without a significant effect of the type of restoration (Kanaan et al. 2026). These findings suggest that, from the patient's perspective, both restorative approaches provided satisfactory functional outcomes in the short‐term. This is particularly relevant in the posterior region, where aesthetic differences may be less perceptible or clinically meaningful to patients. The absence of differences in patient satisfaction indicates that clinical parameters that favour indirect restorations do not necessarily translate into superior patient‐perceived outcomes. This supports the interpretation that patient‐reported outcomes should be regarded as complementary to, rather than interchangeable with, clinician‐rated restoration quality. This is also consistent with recent restorative decision‐making concepts, in which material selection is considered to depend not only on clinical and material‐related factors, but also on patient‐related factors such as costs, acceptance of possible reintervention, aesthetic expectations, age, and functional risk (Manolakis 2026).

The current study has several limitations. Patient inclusion was not limited to those with low caries risk. This potentially introduces more failures due to caries in the follow‐up. However, in clinical practice, patients with heavily restored, endodontically treated molars often present with moderate or high caries risk, or a history thereof. The extended recruitment period of 8 years, which was necessary to reach the required sample size in a narrowly defined patient population, may be viewed as another potential limitation. However, the risk of variability due to temporal factors was minimized by the strict adherence to a standardized clinical protocol throughout the study. All restorative procedures were performed by a single experienced operator using the same materials, adhesive protocols, and curing lights as outlined at the study's outset. Despite the lengthy inclusion period, it reflects the pragmatic realities of conducting randomized controlled trials in real‐world dental settings. Although direct resin composite restorations showed favourable 24‐month survival in the present study, these findings should be interpreted with caution, since this modality does not represent a definitive treatment option for all severely structurally compromised endodontically treated molars. The restorations were placed under strict conditions, including adequate isolation, cuspal coverage, and treatment by an experienced operator. Therefore, direct resin composite restorations in molars with one or no remaining walls should be considered only in carefully selected patients, for example when financial constraints, patient preference, or the need for an immediate restoration influence treatment planning. In such cases, caries and occlusal risk control, patient compliance, and close follow‐up are essential. Longer‐term follow‐up is needed to determine whether the favourable short‐term outcomes of direct resin composite restorations in severely compromised molar teeth are maintained over time.

This randomized controlled trial demonstrated that both direct microhybrid resin composite restorations and lithium disilicate endocrowns are effective treatment modalities for the restoration of severely compromised, endodontically treated molars over a 24‐month period. Restoration and abutment tooth survival and success were high in both groups, with no statistically significant differences observed. While lithium disilicate endocrowns showed superior performance in surface lustre, wear resistance, and mucosal health, direct resin composite offered comparable survival outcomes and patient satisfaction. Further research involving extended follow‐up durations and larger cohorts is warranted to elucidate whether the early clinical differences observed translate into meaningful long‐term variations in restoration performance or the need for retreatment.

## 5 Conclusion

Within the limitations of this clinical trial, both restoration types performed acceptably in restoring severely structurally compromised endodontically treated molar teeth after 24 months. Lithium disilicate endocrowns showed better performance in surface lustre, wear resistance, and mucosal health. However, these qualitative differences did not translate into differences in patient satisfaction, indicating that both restorative options were perceived as equally acceptable by patients in the short‐term.

## Author Contributions

Conceptualization: Maurits C.F.M. de Kuijper, Marco M.M. Gresnigt, Marco S. Cune. Methodology: Maurits C.F.M. de Kuijper, Marco M.M. Gresnigt, Marco S. Cune. Software: Maurits C.F.M. de Kuijper. Validation: Maurits C.F.M. de Kuijper, Carline R.G. van den Breemer, Yvonne C.M. de Waal, Marco M.M. Gresnigt, Marco S. Cune. Formal analysis: Lieke Lanjouw. Investigation: Maurits C.F.M. de Kuijper, Carline R.G. van den Breemer, Yvonne C.M. de Waal. Resources: Maurits C.F.M. de Kuijper, Carline R.G. van den Breemer, Yvonne C.M. de Waal, Marco M.M. Gresnigt, Marco S. Cune. Data curation: Maurits C.F.M. de Kuijper, Carline R.G. van den Breemer, Yvonne C.M. de Waal, Lieke Lanjouw. Writing – original draft: Maurits C.F.M. de Kuijper, Carline R.G. van den Breemer, Yvonne C.M. de Waal, Mutlu Özcan, Marco M.M. Gresnigt, Marco S. Cune. Writing – review and editing: Maurits C.F.M. de Kuijper, Carline R.G. van den Breemer, Yvonne C.M. de Waal, Mutlu Özcan, Marco M.M. Gresnigt, Marco S. Cune, Lieke Lanjouw. Visualization: Maurits C.F.M. de Kuijper. Supervision: Marco M.M. Gresnigt, Marco S. Cune, Mutlu Özcan. Project administration: Maurits C.F.M. de Kuijper, Carline R.G. van den Breemer, Yvonne C.M. de Waal.

## Funding

This study was supported by a research grant of the Dutch society for dental science, Stichting Bevordering Tandheelkundige Kennis (grant number 2015).

## Conflicts of Interest

The authors declare no conflicts of interest.

## Supporting information


**Table S1:** Distributions of the caries risk and presence of parafunction for patients that received 2 endocrown restorations.

## Data Availability

The data that support the findings of this study are available from the corresponding author upon reasonable request.
