# Supplementary material for: Direct Resin Composite and Lithium Disilicate Endocrowns in Severely Compromised Endodontically Treated Molars: A Two‐Year Randomized Controlled Trial
Source: Int Endod J. 2026 Jul 27;59(11):2383–401. doi: 10.1111/iej.70232 (PMC13674819; doi:10.1111/iej.70232)
Supplement: Supplementary file 1 [file IEJ-59-2383-s001.docx]

|  | n (%) |
| --- | --- |
| Caries risk  *Low*  *Medium*  *High* | 4 (24%)  4 (24%)  9 (54%) |
| Parafunction  *No*  *Yes* | 12 (71%)  5 (29%) |
|  |  |

**SUPPLEMENTAL Table 1.** Distributions of the caries risk and presence of parafunction for patients that received 2 endocrown restorations.
